# The GridCAT: A Toolbox for Automated Analysis of Human Grid Cell Codes in fMRI

**DOI:** 10.3389/fninf.2017.00047

**Published:** 2017-07-21

**Authors:** Matthias Stangl, Jonathan Shine, Thomas Wolbers

**Affiliations:** ^1^German Center for Neurodegenerative Diseases (DZNE), Aging and Cognition Research Group Magdeburg, Germany; ^2^Center for Behavioral Brain Sciences Magdeburg, Germany

**Keywords:** grid cells, human, fMRI, general linear model, toolbox, open-source

## Abstract

Human functional magnetic resonance imaging (fMRI) studies examining the putative firing of grid cells (i.e., the grid code) suggest that this cellular mechanism supports not only spatial navigation, but also more abstract cognitive processes. Despite increased interest in this research, there remain relatively few human grid code studies, perhaps due to the complex analysis methods, which are not included in standard fMRI analysis packages. To overcome this, we have developed the Matlab-based open-source Grid Code Analysis Toolbox (GridCAT), which performs all analyses, from the estimation and fitting of the grid code in the general linear model (GLM), to the generation of grid code metrics and plots. The GridCAT, therefore, opens up this cutting-edge research area by allowing users to analyze data by means of a simple and user-friendly graphical user interface (GUI). Researchers confident with programming can edit the open-source code and use example scripts accompanying the GridCAT to implement their own analysis pipelines. Here, we review the current literature in the field of fMRI grid code research with particular focus on the different analysis options that have been implemented, which we describe in detail. Key features of the GridCAT are demonstrated via analysis of an example dataset, which is also provided online together with a detailed manual, so that users can replicate the results presented here, and explore the GridCAT’s functionality. By making the GridCAT available to the wider neuroscience community, we believe that it will prove invaluable in elucidating the role of grid codes in higher-order cognitive processes.

## Introduction

Identifying the neural mechanisms supporting spatial navigation remains a key goal for neuroscience. In recent years, significant progress has been made with the discovery of the grid cell in the rat medial entorhinal cortex, a neuron exhibiting firing properties that could provide a spatial metric underlying navigational functions such as path integration (Hafting et al., 2005). Grid cells have been found subsequently in a diverse range of mammalian species (for a detailed review, see Rowland et al., 2016), and, more recently, the putative signature of grid cell firing, which we refer to as the grid code throughout this article, has been identified also in healthy human subjects using functional magnetic resonance imaging (fMRI; Doeller et al., 2010; Kunz et al., 2015; Constantinescu et al., 2016; Horner et al., 2016). Given the increasing interest in the role of grid cells in human cognition, and the absence of standard analysis tools to examine grid codes in fMRI, we present here the Matlab-based Grid Code Analysis Toolbox (GridCAT), which generates grid code metrics from functional neuroimaging data. The GridCAT is openly available at the Neuroimaging Informatics Tools and Resources Clearinghouse (NITRC) and can be downloaded from: http://www.nitrc.org/projects/gridcat.

Unlike place cells that have single firing fields (O’Keefe and Dostrovsky, 1971), grid cells in the rat medial entorhinal cortex fire in multiple different locations within the environment (Fyhn et al., 2004; Hafting et al., 2005). The firing fields of these cells show remarkably regular organization, forming tessellating equilateral triangles that effectively “tile” the world’s navigable surface in a hexagonal lattice (Hafting et al., 2005). The equally spaced and repetitive firing means that, for each firing field of a grid cell, the six adjacent fields are arranged in 60° intervals, creating a six-fold symmetry. Each cell’s grid can differ, however, in several ways, such as its orientation, spacing and the size of its firing field. Although recording from a population of grid cells within the entorhinal cortex reveals variability in these properties, there appears to be topographical arrangement of these cells. For example, the grids of neighboring cells are more similar in terms of their orientation and spatial scale relative to cells located further apart (Hafting et al., 2005); distal cells, however, can still show coherence in the orientation of their grids, even though their spatial scales may differ (Barry et al., 2007). Given that each cell’s grid is spatially offset (to varying degrees) relative to a neighboring one, it has been hypothesized that these cells may provide the neural mechanism for complex spatial navigation abilities such as path integration (Hafting et al., 2005).

Grid cells have been found in a number of other species, including bats (Yartsev et al., 2011), primates (Killian et al., 2012) and humans (Jacobs et al., 2013). Although rare and often comprising small sample sizes, studies using intracranial recordings in humans provide an opportunity to use experimental methods analogous to those routinely used in behavioral neuroscience (i.e., recording directly from neurons). Jacobs et al. (2013) recorded from cells in the entorhinal cortex of patients with intractable epilepsy as they completed an object-place memory task requiring them to navigate a virtual environment. Consistent with the rat electrophysiology, there was evidence of cells with a six-fold symmetry in their firing rate. The grid cell, therefore, appears preserved across different mammalian species, including humans, and may comprise a common neural mechanism for spatial navigation.

**Figure 1:**
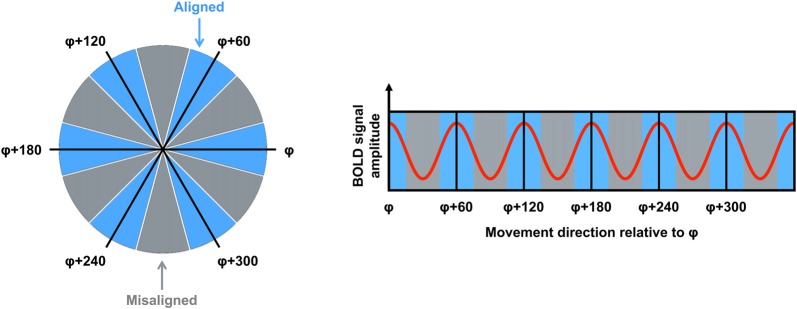
The logic for grid code analysis in human functional magnetic resonance imaging (fMRI). Left: movement directions can be categorized either as aligned (blue) or misaligned (gray) with the mean grid orientation (φ). Right: the red curve shows the expected pattern of the blood oxygenation level dependent (BOLD) signal modulated by movement direction relative to φ. High signal peaks are expected for movements aligned with φ or a 60° multiple of φ (blue sectors). Figure adapted from Doeller et al. (2010).

fMRI is used commonly to investigate the neural correlates of higher-order cognitive processes in large samples of healthy subjects, and this method has been applied to the study of putative grid cell firing (Doeller et al., 2010). Although fMRI is able only to detect changes in signal over thousands of neurons, several properties of grid cell firing suggested it would be possible to detect grid codes in the blood oxygenation level dependent (BOLD) response at the macroscopic level. First, as described earlier, even though grid cells are arranged topographically, the grid orientation of distal cells may still be coherent (Barry et al., 2007). This means that the global signal associated with the firing of these neurons would be consistent both within a voxel and across different voxels. Second, the firing rate of a subpopulation of grid cells, known as conjunctive grid cells, is further modulated by the animal’s movement direction in the environment. Specifically, the firing rate of conjunctive grid cells is increased when the animal travels in the cell’s “preferred” direction relative to other travel directions (Sargolini et al., 2006). Furthermore, the preferred firing direction of conjunctive grid cells is aligned with the main axes of the grid (Doeller et al., 2010). Together, these differences in the dynamics of grid cell firing could be reflected in a six-fold sinusoidal pattern observable in the BOLD response (i.e., the grid code) when participants performed translations either aligned or misaligned with the grid’s axes (Figure 1). Using an object-place memory task in a virtual environment, Doeller et al. (2010) found exactly this pattern of data in several brain regions, including the entorhinal cortex. Consistent with the results of rodent electrophysiology, the BOLD signal showed a six-fold symmetry, with greater activity associated with translations in which the travel direction was aligned with the mean grid orientation, compared to when the travel path was misaligned with a grid axis (the precise methods for estimating the mean grid orientation, and testing the model, are described in detail below). This study, therefore, was critical in demonstrating that fMRI could be used to study grid codes in humans.

The identification of grid codes using fMRI has already generated a number of promising new research questions. For example, the estimation of grid codes has been shown to have potential clinical applications with reduced grid code magnitude evident in those at increased genetic risk of Alzheimer’s disease (Kunz et al., 2015). Furthermore, although there appear to be commonalities in the neural mechanisms supporting navigation across diverse species, the study of grid codes in fMRI has demonstrated that these spatial codes may be used more flexibly in humans (Horner et al., 2016). Specifically, Horner et al. (2016) found evidence of the sinusoidal pattern in the BOLD response when participants imagined navigation in a virtual environment, despite the absence of visual input. Finally, Constantinescu et al. (2016) demonstrated that recently acquired conceptual knowledge is organized using the same six-fold spatial symmetry. In humans, therefore, these grid codes may be used more abstractly in service of higher-order cognitive processes beyond pure spatial navigation.

**Figure 2:**
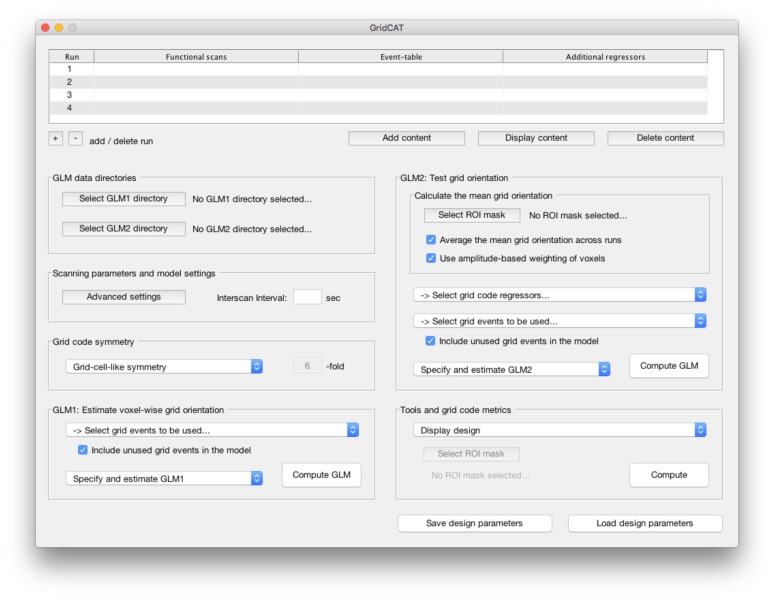
The graphical user interface (GUI) of the Grid Code Analysis Toolbox (GridCAT). A grid code analysis can be carried out via the GUI by specifying data, parameters and settings, depending on the individual experimental design and research question. The GUI offers all analysis options of the grid code analysis pipeline as well as a set of additional tools to generate grid code metrics, visualize results and export the resulting data. A detailed explanation of all GUI options and how to use all functions of the GridCAT via the GUI, is provided in the GridCAT user manual that is distributed along with the open-source code. Please note that the visual appearance of the GUI might differ between operating systems and versions of Matlab.

**Figure 3:**
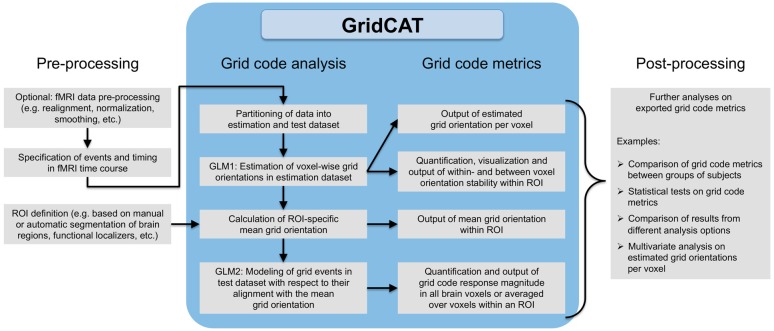
Grid code analysis pipeline. All that is required to perform a grid code analysis are functional brain images (which, depending on the user’s wishes, may have undergone standard fMRI data preprocessing such as smoothing etc.) together with a file detailing events of interest during the fMRI time course and their corresponding timing information. The GridCAT partitions these data into an estimation dataset and a test dataset, offering multiple options as to how to split the data depending on the experimental design and the user’s needs. Using the estimation dataset, the GridCAT then estimates voxel-wise grid orientations of the grid code in a first general linear model (GLM1). As a result, voxel-wise grid orientations are stored and can be plotted using the GridCAT’s specific plotting options to visualize grid code stability both within and between voxels, or can be exported in several formats for further analysis such as group level analyses, statistical testing, or multivariate analysis methods. Moreover, within any region of interest (ROI), the GridCAT can calculate an ROI-specific mean grid orientation, providing that the mask image (e.g., anatomically or functionally defined) and functional data are registered to one another. Finally, in a second GLM (GLM2) the GridCAT allows events in the test dataset to be modeled with respect to their alignment with the ROI-specific mean grid orientation, in order to quantify the grid code response magnitude individually for all brain voxels or averaged over voxels within an ROI. All results and grid code metrics can be exported for further use with statistical and neuroimaging analysis tools of the researcher’s choice.

Grid code analyses are distinctly non-trivial, requiring a range of skills, including computer programming, and knowledge of specific mathematical techniques (e.g., quadrature filter techniques). Not all cognitive neuroscientists who wish to examine cognitive processes related to grid cell firing in humans possess these skills. To cater for these researchers, the GridCAT provides a simple graphical user interface (GUI; Figure 2), meaning that the user is not required to work directly with the source code. Moreover, given that no standard analysis package offers the necessary algorithms to detect grid codes in fMRI data, even researchers who are capable of reproducing all necessary analysis steps may find it a demanding and time-consuming task to write the source code required for this type of analysis. The GridCAT addresses these issues by performing automatically all steps in the grid code analysis pipeline (as summarized in Figure 3). By removing the need to develop source code independently, the toolbox opens up this exciting research area to the wider neuroscience community, and saves researchers time, allowing them to address novel research questions regarding the role of grid cells in human cognition.

A further aim of this article is to provide, for the first time, a comprehensive overview of the different analysis strategies that have been used to date. By synthesizing these different approaches, we hope to inform researchers who are new to the field about the different possible ways in which the fMRI data can be modeled to assess grid code metrics. Finally, by making a number of analysis options available in the toolbox, the GridCAT will also help to standardize analyses across the research community, making data analysis pipelines more comparable across different labs, and stimulating discussion in this exciting and rapidly developing research area.

The GridCAT allows users to input easily their study design and performs all analyses to estimate the grid code in functional images and generates automatically grid code metrics. Results can be visualized using built-in plotting functions and the data exported for further analysis depending on the user’s needs. It requires only a basic Matlab installation (i.e., no additional Mathworks toolboxes are required) and SPM12^1^, and is compatible with Windows, Linux and Mac OS. A detailed manual guides users through all steps of a grid code analysis and an example dataset is provided with the GridCAT to explore its functionality.

Although there are similarities across fMRI studies in the methods used to estimate grid code metrics, there is as yet no standard analysis pipeline. Because of this, the GridCAT has been designed to be flexible in accommodating a number of different analysis options; decisions regarding a researcher’s own pipeline will depend upon paradigm-specifics and the research question of interest. We note that a recent grid code study examined the neural signal associated with imagined trajectories in the environment (Bellmund et al., 2016). We do not discuss this experiment here, however, because, rather than the mass univariate method commonly used in the study of grid codes, they used multivariate representational similarity analysis, for which there are already several toolboxes available (e.g., Nili et al., 2014; Oosterhof et al., 2016). In the following section, we review extant methods for deriving grid codes in fMRI, and highlight differences in analysis approaches. The aim of this review is to inform the GridCAT user of the different analysis options that have been used previously, and that are available in the toolbox, rather than to provide a critique as to best practice for deriving grid code metrics.

## Grid Code Analysis

Although analysis pipelines for the examination of grid codes using fMRI differ in several aspects, the overall procedure is relatively similar. First, events of interest for the grid code analysis (i.e., grid events) are specified in the time course of the imaging data. Second, the imaging data are then partitioned into estimation and test datasets. Third, a general linear model (GLM) is fit to the estimation dataset to estimate voxel-wise orientations of the grid code (i.e., GLM1). Fourth, these voxel-wise orientation values are then averaged over voxels in a region of interest (ROI) to generate a mean grid orientation used for a second GLM in which grid events of the test dataset are modeled with respect to their alignment with the mean grid orientation (i.e., GLM2). Finally, grid code metrics are computed, such as the magnitude of grid code response as well as measures of between- or within-voxel orientation coherence of the grid code. In the following sections, we provide more information regarding these individual steps of the grid code analysis pipeline (see also Figure 3 for a comprehensive overview).

### Functional Image Preprocessing for Grid Code Analysis

The GridCAT is agnostic with regards to the nature of the preprocessing carried out on functional images prior to the grid code analysis. For example, the analysis can be conducted using a participant’s normalized, and smoothed, functional images (Doeller et al., 2010; Constantinescu et al., 2016; Horner et al., 2016). Alternatively, one could work in the individual subject’s native functional space (Kunz et al., 2015). Motivations for normalizing to standard space prior to analysis include the desire to examine group-level, cluster-statistics (e.g., Constantinescu et al., 2016), whereas researchers concerned about spatial distortions or interpolation errors in their data resulting from normalization to a standard template might choose to perform the grid code analysis in the participant’s native space. fMRI preprocessing can be carried out in the researcher’s neuroimaging analysis package of choice.

### Specifying Grid Events

Before the grid code can be estimated, it is necessary to specify grid events within the fMRI time course. For example, grid events could comprise periods of translational movement (e.g., Doeller et al., 2010; Kunz et al., 2015; Horner et al., 2016) within a virtual environment. For each grid event, an angle relative to a nominal 0° reference point (e.g., a fixed landmark in the virtual environment) is then defined, resulting in the “grid event angle”. More details as to how grid events are defined for use in the GridCAT analysis pipeline are provided in the GridCAT manual.

### Partitioning the Grid Code Data into Estimation and Test Sets

Given that the functional data are labeled either as estimation or test data, researchers must decide how to perform this partition. One method is to split the data run-wise into odd and even runs (Doeller et al., 2010; Kunz et al., 2015), performing the estimation in the odd runs and testing in the even ones (or vice-versa). Alternatively, one could split the data into *n* temporal bins, and perform the same analysis on these odd/even bins (Horner et al., 2016). As well as offering these data partitioning methods, the GridCAT also provides options to separate grid events within each scanning run into odd and even events, or to split each scanning run into two halves so that estimation and test are calculated on the first and second halves of runs, respectively. Furthermore, if these default partitioning options are not suitable for a particular experiment, bespoke partitioning schemes can be specified in the GridCAT event-table (which is described in detail in the GridCAT manual), allowing the user to specify whether a particular grid event should be assigned either to the estimation or test dataset.

### Estimating Grid Orientations in the BOLD Signal

For the estimation data (GLM1), the grid event angle is used to create two parametric regressors for the grid events, using sin(α_t_*6) and cos(α_t_*6), respectively, where α_t_ represents the grid event angle. The multiplication term (*6) used in the calculation of these two regressors transforms the grid event angle into 60° space, mirroring the hexagonal symmetry observed in grid cell firing. By including these parametric regressors in the GLM, voxels with time courses showing modulation of their signal according to six evenly spaced 60° intervals would have parameter estimates (i.e., beta weights that have been estimated for a regressor in the GLM, with higher parameter estimates indicating a better model fit) with high absolute amplitudes. When calculating GLM1, the GridCAT allows users to include additional regressors (e.g., nuisance regressors, such as movement parameters), add time and dispersion derivatives of the hemodynamic response function, and change modeling parameters (e.g., high-pass filtering, microtime onset and resolution, masking threshold), depending on the individual experimental design. More details about these options can be found in the GridCAT manual.

Voxel-wise grid orientations resulting from GLM1 can be visualized using the GridCAT’s specific plotting options, and different grid code metrics such as grid code stability both within voxels (e.g., over time) and between voxels (e.g., coherence of grid orientations within an ROI) can be calculated (see “Analysis of Grid Code Stability” Section). Plots can be saved in different file-formats, so that users can subsequently load them into any image processing software and adapt their visual appearance, depending on individual needs. For further analysis such as group level analyses, statistical testing, or multivariate analysis methods on voxel-wise grid orientations, these data can be exported in several formats (e.g., as a data vector, or as a 3D NIfTI image).

Following GLM1, the GridCAT can then calculate the mean grid orientation across all voxels in an ROI. To compute the mean grid orientation, the beta estimates (β1 and β2) associated with the two parametric regressors are each averaged over all voxels in the ROI, and the resulting two values submitted to: arctan[mean(β1)/mean(β2)]/6. Once the mean grid orientation has been calculated, this value can be used to categorize individual grid event angles in the test data (GLM2) to determine the magnitude of the grid code response. For example, grid event angles could be classified either as aligned or misaligned with the mean grid orientation (see “Quantifying the Magnitude of the Grid Code Response” Section).

### ROI Selection

As described above, the mean grid orientation can be calculated in any chosen ROI, providing that the mask and functional data are registered to one another. Popular choices of ROI include anatomical masks, such as the entorhinal cortex (Doeller et al., 2010; Kunz et al., 2015; Horner et al., 2016), however it is possible also to input to the GridCAT a functionally-defined mask from an orthogonal contrast (e.g., Constantinescu et al., 2016), or localizer dataset.

### Quantifying the Magnitude of the Grid Code Response

The greatest degree of heterogeneity in analysis pipelines of fMRI grid code studies stems from how the grid code is quantified, or the choice of grid code metric. This relates, in part, to the research question of interest, and we outline here the different methods that have been used thus far in the published literature. It is worth noting that these methods are not mutually exclusive, and a researcher may want to use a combination of different approaches to test a number of different hypotheses.

In the following sections (“Parametric Modulation” and “Comparing Activity Associated with Aligned vs. Misaligned Events”), we describe different methods to set-up GLM2 where grid events are modeled with respect to their alignment with the ROI-specific mean grid orientation. Irrespective of the method used, additional regressors can be added to GLM2, and modeling parameters can be changed depending on the individual experimental design (as described for GLM1).

Following GLM2, estimates of the grid code response magnitude can be exported either as a 3D NIfTI image containing estimates for all individual brain voxels or as an average of the grid code response magnitude within an ROI, so that researchers can conduct further analyses on these data using statistical or neuroimaging data analysis and visualization tools of their choice.

#### Parametric Modulation

In the original study reporting grid codes in the fMRI signal, Doeller et al. (2010) fitted a parametric regressor to the grid events in the test data to examine whether voxels in an entorhinal cortex ROI showed evidence of a six-fold sinusoidal pattern of activity. The parametric regressor was calculated by taking each grid event angle (α_t_), and determining its difference from the mean grid orientation (φ) by calculating cos[6*(α_t_ – φ)], which resulted in values ranging between “1”, for grid event angles aligned perfectly with the mean grid orientation (or a 60° multiple of it), and “−1” for values completely misaligned with the grid code phase (i.e., mean grid orientation +30°, plus any 60° multiple of this value). Using cluster statistics, Doeller et al. (2010) reported voxels at the group-level showing modulation of their signal according to this sinusoidal function. A similar analysis was used in Horner et al. (2016), with the exception that they used a contrast to look for brain regions in which the sinusoidal model fits significantly better for one condition vs. another (i.e., imagined navigation vs. stationary periods).

#### Comparing Activity Associated with Aligned vs. Misaligned Events

It is possible also to compare parameter estimates associated with aligned vs. misaligned grid events. For example, in a subsequent analysis, Doeller et al. (2010) separated grid events into two regressors comprising those translations aligned within 15° of a grid axis vs. those more than 15° from a grid axis, and again showed that significantly greater activity in entorhinal cortex was associated with events aligned with grid axes. This analysis strategy was used also by Kunz et al. (2015) who found that participants at increased genetic risk of Alzheimer’s disease show reduced BOLD response, relative to control participants, when contrasting trials “aligned > misaligned” with the grid axis (i.e., a reduction in the ability to detect the grid code). Constantinescu et al. (2016) used a variation of the aligned vs. misaligned analysis by sorting the grid event angles into 12 different regressors, each representing a 30° bin. Six regressors comprised aligned trials, those events within ±15° of the mean grid orientation (or a 60° multiple of it). The remaining six regressors comprised misaligned trials, that is events offset from the mean grid orientation by 30° (plus a 60° multiple of this value) ±15°, and parameter estimates were extracted for each regressor.

### Analysis of Grid Code Stability

The ability to detect the grid code in fMRI can be affected by the stability of the estimated grid orientation either between voxels within an ROI, or within voxels across different scanning runs and/or conditions (e.g., stability over time or different spatial environments, respectively). In terms of grid orientation stability between voxels within an ROI, if all voxels provide a different orientation value, then the resulting mean grid orientation would be random, and the coding of grid events in the test data depending on their deviation from the mean grid orientation would be arbitrary. To test whether there was evidence of coherence in the orientation of the grid code between different voxels in their entorhinal cortex ROI, Doeller et al. (2010) submitted all voxel orientation values to Rayleigh’s test for non-uniformity of circular data. Doeller et al. (2010) reported significant clustering of estimated orientations in around three-quarters of their participants.

Alternatively, an inability to detect grid codes in the fMRI signal could result from instability of the estimated grid orientation within a voxel over time. Kunz et al. (2015) tested the stability of the grid orientation over time by extracting the orientation of a voxel in one half of the data and comparing this to the same voxel’s orientation in the second half of the data. These data were scored such that if the values were within ±15° of one another, then the grid orientation for the voxel was classified as stable. At the ROI level, the percentage of voxels showing stability in their estimated orientation over time could then be calculated. Even though participants at risk of Alzheimer’s disease showed coherence in grid orientation between voxels within a single scanning run, over time the orientation estimates for a given voxel differed. It was concluded, therefore, that the reduced ability to detect grid codes in the risk group resulted from instability in the orientation within-, but not between-, voxels in the Alzheimer’s risk group.

### Control Analyses

Given that grid cells identified in rodents show a strict six-fold symmetry in their firing, it is necessary to test whether the best fit for the grid code analysis in fMRI is also a six-fold model, or whether other sinusoidal models fit the data equally well. In all studies published to date, the six-fold model has proven a better fit to estimate the orientation of the grid code in comparison to other symmetrical models (three-, four-, five-, seven- and eight-fold models; Doeller et al., 2010; Kunz et al., 2015; Constantinescu et al., 2016; Horner et al., 2016). These different models can be implemented in the GridCAT, allowing the user to examine whether the six-fold model provides a better fit to the data.

An alternative control analysis, which can be carried out using the GridCAT, is to test for the grid code in regions where one would not expect to observe this signal (e.g., the visual cortex). Although this type of control analysis has been used previously (Doeller et al., 2010), it may be difficult to predict exactly where in the brain one would expect to see this pattern of data. For example, using an orthogonal localizer contrast, Constantinescu et al. (2016) found evidence of the sinusoidal response in a number of different regions including the ventromedial prefrontal cortex, and the posterior cingulate cortex.

## Analysis of Example Dataset

To demonstrate some of the key features of the GridCAT, we detail here the analysis of functional data from an example participant who was scanned whilst completing a spatial navigation task. The dataset of this example participant is available for download, so that the complete analysis pipeline described here can be reproduced using the GridCAT, giving the user the opportunity to explore its tools and functions. Furthermore, all necessary steps to analyze the example dataset are described in detail in the GridCAT manual.

### Methods

The example participant was 28 years old, right handed, had normal vision and no history of psychiatric or neurological disorders. Informed consent was obtained in writing in accordance with the Declaration of Helsinki before the measurements, and the experiment received approval from the Ethics Committee of the Otto-von-Guericke University of Magdeburg.

#### Spatial Navigation Task

**Figure 4:**
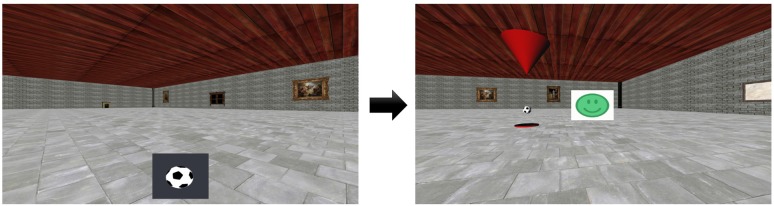
Example trial during fMRI scanning. Prior to scanning, the participant learned the locations of three target-objects in a virtual environment. Left: during scanning, one of the target-objects was cued (e.g., a football) and the participant was asked to navigate to its location. Right: after the participant pressed a button to confirm their choice of location, the target-object appeared at its correct location and a smiley-face provided feedback as to the accuracy of the response.

Prior to scanning, the participant was asked to navigate a square virtual space (160 × 160 virtual meters) using a joystick and learn the location of three target-objects. Afterwards, the participant underwent two separate runs of fMRI scanning during which the participant navigated in the same virtual space. Each trial had the following structure: at the start, all target-objects disappeared and an image of one of them was shown at the bottom of the screen (Figure 4). The participant was asked to navigate to the position of the cued target-object and confirm their choice of location with a button-press. After the button-press, feedback was given to the participant via the target-object appearing at its correct location and a smiley-face displayed on the screen that was either green (if the “error distance” between the correct location and the participant’s response was below 20 virtual meters), yellow (for “error distances” between 20 and 30 virtual meters), or red (for “error distances” larger than 30 virtual meters). After each trial, the participant was automatically transported to a random position within the virtual space. Each scanning run lasted 16 min, and the participant was asked to complete as many trials as possible.

#### Scanning Parameters

T2*-weighted functional images were acquired on a 3T Siemens Magnetom Prisma scanner using a partial-volume echo-planar imaging (EPI) sequence with the following parameters: repetition time (TR) = 1500 ms, echo time (TE) = 30 ms, slice thickness = 2 mm, in-plane-resolution = 2 × 2 mm, number of slices = 24, field of view = 216 mm, flip angle = 80°, slice acquisition order = interleaved.

For manual delineation of the entorhinal cortex, a high-resolution T2-weighted structural image was acquired using a turbo-spin-echo (TSE) sequence with the following parameters: TR = 6000 ms, TE = 71 ms, slice thickness = 2 mm, in-plane-resolution = 0.5 × 0.5 mm, number of slices = 64, field of view = 224 mm, flip angle = 120°, slice acquisition order = interleaved.

#### Analysis Pipeline

Prior to analyses using the GridCAT, the functional images for the two runs were realigned and smoothed (5 mm FWHM) using SPM12. Anatomical masks of the right and left entorhinal cortices were traced manually (following Ding et al., 2016) on the participant’s T2-weighted image using ITK-SNAP^2^, and co-registered to the EPI data. These two anatomical masks were used as separate ROIs for all following analyses.

As detailed in the previous sections, there are a number of different ways grid codes can be examined in fMRI data, which are available to the GridCAT user. It is beyond the scope of this article to demonstrate all possible combinations of modeling options; therefore, we chose a subset of parameters for the grid code analysis detailed here. The first parameter relates to the way in which the mean grid orientation is calculated. In GLM1, the GridCAT generates an image containing voxel-wise grid orientations, which can then be used to determine the mean grid orientation for a given ROI. The mean grid orientation can be calculated by averaging over voxels in the ROI either within individual scanning runs, or across multiple runs. For example, if one predicts that the grid orientation will change over runs, perhaps due to an experimental manipulation that could induce grid cell remapping (Fyhn et al., 2007), it would be sensible to estimate the grid orientation within individual runs, rather than averaging across them. Although we did not predict that there would be any changes in grid orientation over the two runs in our paradigm, we demonstrate the effect of estimating the mean grid orientation within vs. across runs.

We examined also two different ways in which the grid events (i.e., translational movements within the virtual environment) can be modeled in GLM2. In one model, grid events were modeled using a single parametric modulator regressor (e.g., Doeller et al., 2010). In an alternate model, replicating the analysis of Kunz et al. (2015), grid events were separated into two regressors — aligned or misaligned with the mean grid orientation — and contrasted with one another (“aligned > misaligned”). The approach used by Constantinescu et al. (2016) in which grid events are separated into 12 different regressors comprising 30° bins was not used here because our paradigm allowed for free exploration of the environment and therefore it is possible that not all directions were sampled equally. In all GLMs, we included as regressors of no interest the feedback phase in the paradigm, head motion parameters (*x*, *y*, *z*, yaw, pitch and roll) derived from realignment in SPM, and the unused grid events (i.e., the grid events for GLM2 when fitting GLM1, and vice-versa).

Finally, we show how different symmetrical models (four-, five-, six-, seven- and eight-fold) affect the model fit, with the prediction that the six-fold symmetrical model should provide the highest parameter estimates, given that this reflects grid cell firing symmetry.

### Results

**Figure 5:**
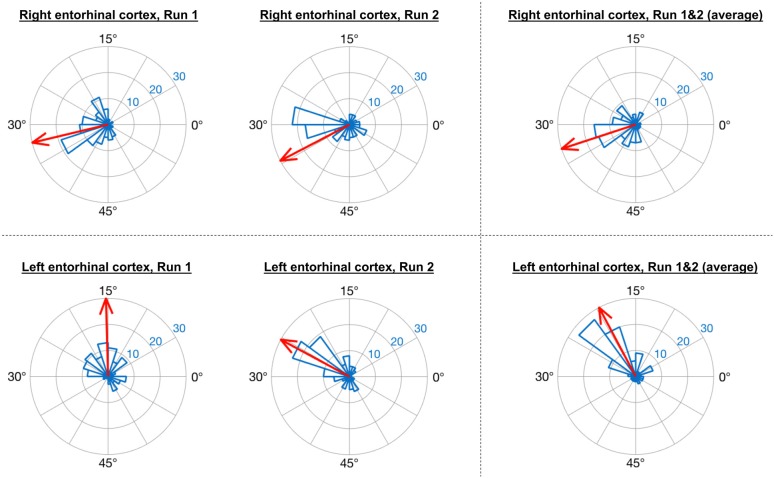
GridCAT polar histogram plots showing coherence of the grid orientation between voxels in right and left entorhinal cortex ROIs. The length of each bar indicates the number of voxels that share a similar grid orientation, and the blue numbers indicate the number of voxels represented by each ring of the polar plot. The GridCAT also allows the user to calculate and visualize the mean grid orientation (red arrow) for each plot (which is used in GLM2 to model grid events with respect to their deviation from the mean grid orientation). Depending on the user’s choice of model, the mean grid orientation can be calculated separately for run 1 (left column) and run 2 (middle column), or alternatively, the mean grid orientation over multiple runs (right column) can be calculated by averaging the parameter estimates. Furthermore, users can choose to carry out Rayleigh’s test for non-uniformity of circular data. Rayleigh’s test indicated that voxels in both the right (top row) and left (bottom row) entorhinal cortex showed significant clustering (i.e., coherence) in their orientations (all *p* < 0.00001).

Consistent with the analysis strategy of Doeller et al. (2010), in GLM1 we found that the orientations of grid codes in voxels of both right and left entorhinal cortex showed significant non-uniformity, or clustering (see Figure 5). The GridCAT produces polar histogram plots, which indicate the different orientations derived from voxels in a given ROI, and the number of voxels sharing similar orientations. In these interactive plots, the mean grid orientation of all voxels within the ROI can also be calculated and plotted by the GridCAT. Moreover, Rayleigh’s test for non-uniformity of circular data can be carried out (applying code from the open-source toolbox CircStat2012a; Berens, 2009), in order to test whether the orientations of the grid code in voxels within an ROI show greater clustering than would be expected by chance. The example data suggest, therefore, that there is stability in grid orientation between voxels within the entorhinal cortex. As can be seen in Figure 5, the voxel-wise orientations estimated in the two separate runs were similar to one another, suggesting that the mean grid orientation could be calculated across both runs and used to categorize grid events in GLM2. If, however, these plots had indicated that the mean grid orientations changed over runs, the user might consider estimating and testing grid orientations within individual runs so that the categorization of grid events in GLM2, according to their alignment with the mean grid orientation, was more accurate. Furthermore, the GridCAT allows for the export of voxel-wise orientation values within an ROI, in order for additional analyses and/or statistical tests to be conducted on these data, depending on the user’s specific research question.

**Figure 6:**
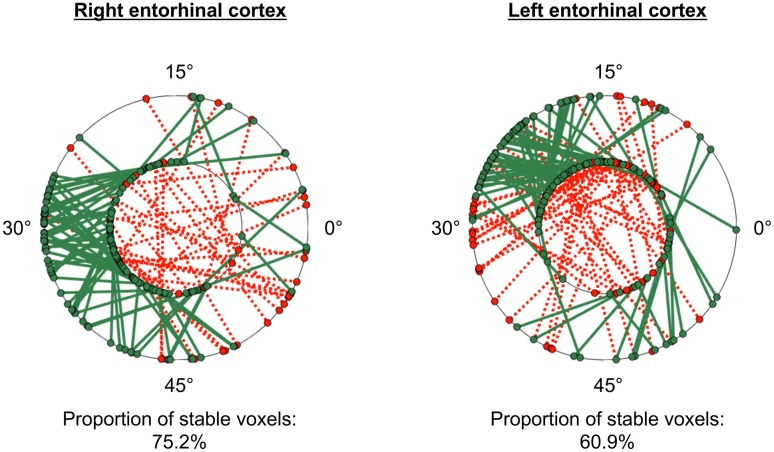
GridCAT polar plots showing coherence of the grid orientation within voxels, across runs 1 and 2, in right and left entorhinal cortex ROIs. In both the right and left entorhinal cortex, the majority of voxels maintained the same grid orientation (±15°) across the two runs; the proportion of voxels maintaining the same orientation across runs is calculated automatically for the user. The two black rings in each plot represent the two different runs (inner ring: run 1, outer ring: run 2), and the orientation of the grid code for each voxel is indicated with a circular marker; a line connects the orientations of each voxel. Green solid lines indicate voxels with stable orientations, whereas red dotted lines indicate voxels with unstable orientations. The GridCAT allows the user also to customize the plots, including the color schemes, line styles, as well as adapting the threshold for classifying a voxel as stable.

The GridCAT can test also the within-voxel stability of the grid orientation across different scanning runs and/or conditions. When the user inputs two different voxel-wise orientation images derived from GLM1, and an ROI, the toolbox generates a plot comprising two polar plot rings (see Figure 6). For the analysis presented here, each ring represents a different scanning run, and circle markers denote the grid orientation of individual voxels; straight lines connect grid orientations of the same voxel across different runs. By default, the orientation of the grid code in a voxel is considered stable if the two values are within ±15° of one another (i.e., the same threshold used in Kunz et al., 2015), and the GridCAT outputs the proportion of voxels within an ROI surviving this threshold. The stability of individual voxels is also displayed via the color of the connecting line; here, the GridCAT has displayed stable voxels in green and unstable voxels in red. Consistent with Kunz et al. (2015), the grid orientation for the example participant was consistent across the two runs, such that 75% of voxels in the right entorhinal cortex, and 60% of voxels in the left entorhinal cortex, maintained a stable orientation. The GridCAT provides the user with several other options in an interactive plot, including the ability to change the threshold value for stability (i.e., ±15°) if the researcher wishes to be more conservative or liberal with this estimate. Moreover, the user can specify several aesthetics of the plot, such as the colors and styles of the lines and markers.

**Figure 7:**
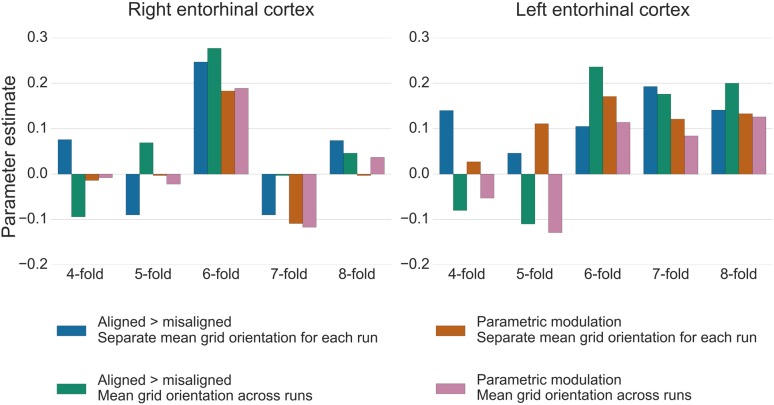
Parameter estimates from GLM2 associated with grid events using different model designs and grid code symmetries. In the right entorhinal cortex, the six-fold symmetrical model provided the best model fit, both for the “aligned > misaligned” contrast and for the single parametric modulation regressor. Calculating the mean grid orientation over two runs, vs. using separate mean grid orientations for each run, made little difference to the parameter estimates. In the left entorhinal cortex, the effect of generating the mean grid orientation over multiple runs vs. separate single runs was more variable and other symmetrical models provided equally good model fits for this hemisphere. All bars show the mean parameter estimate averaged over all voxels within right and left entorhinal cortex, respectively.

For the test data in GLM2, the GridCAT allows users to model grid events either with a parametric modulator regressor (e.g., Doeller et al., 2010), or by separating grid events into trials aligned vs. misaligned with the mean grid orientation and contrasting these values (“aligned > misaligned”). The two methods resulted in comparable parameter estimates in the right entorhinal cortex ROI, with the “aligned > misaligned” contrast method associated with slightly higher parameter estimates relative to the parametric modulator (see Figure 7). In the left entorhinal cortex, there were less obvious differences between methods, however the “aligned > misaligned” contrast again yielded the highest parameter estimate, but only when the mean average grid orientation was calculated using the data from both runs in GLM1. That the grid code metrics appear generally stronger in the right hemisphere, in terms of between-voxel and within-voxel grid orientation coherence, and model fit in GLM2, supports previous findings (Doeller et al., 2010). It is unclear from a theoretical viewpoint, however, why this should be the case, and requires more extensive comparisons within individual subjects to determine the consistency of this effect.

The control analysis tests for the fit of different symmetrical models, to examine whether the six-fold symmetry explains best the data. Consistent with the other results reported here, in right entorhinal cortex the six-fold symmetrical model resulted in the numerically highest parameter estimates relative to all other models. In the left entorhinal cortex, the six-, seven- and eight-fold models all appear to fit the data equally well (Figure 7). It should be noted, however, that other articles reporting a better fit of the six-fold symmetrical model show effects at the group-level, rather than within individual subjects. Accordingly, there may be substantial variability in these estimates both inter-subject, as well as intra-subject, as demonstrated here by the difference between right and left hemispheres.

## Discussion

The GridCAT is an open-source toolbox allowing researchers to examine the putative firing of grid cells (i.e., the grid code) in human fMRI data. The GridCAT provides a simple and user-friendly GUI, and accompanying open-source code, for the analysis of fMRI data, so that the user can conduct the entire grid code analysis pipeline. In order to learn and understand the functionality of the GridCAT, a detailed manual is provided to guide the user through all analysis steps, and the user can also follow the instructions to analyze an example dataset and reproduce the results presented here. Furthermore, example scripts are provided for those who do not want to use the GUI, but rather use and modify the existing open-source code of the GridCAT. The Support section of NITRC also provides a platform for discussion of issues relating to the toolbox, as well as the opportunity for users to submit any requests or report errors regarding the GridCAT.

Despite the great deal of research into grid cells using non-human animal species (for an overview see Rowland et al., 2016), there remain very few studies examining grid codes in human fMRI. Given that this cellular mechanism is now purported to support more than just pure spatial navigation behavior both in humans (Constantinescu et al., 2016) and rats (Aronov et al., 2017), researchers now face the exciting challenge of elucidating exactly what role this cell type may play in other cognitive domains.

In humans, the architecture of grid cells is unknown, and it remains unclear whether there are multiple different grid codes (derived from the fMRI signal) that represent different types of information across the brain. For example, in terms of spatial navigation, in rodents there is evidence that grid cells are arranged in different modules, with neurons within a module sharing a similar firing amplitude, preferred orientation and spatial scale (Stensola et al., 2012). Although we can only study changes in signal at the macroscopic-level using fMRI, analysis of higher resolution imaging data, which would be supported also by the GridCAT, may reveal heterogeneity of the grid code within a single ROI (reflecting these different properties of grid cell modules). Furthermore, voxels showing a sinusoidal pattern in the BOLD signal have been identified across the brain in human fMRI studies (e.g., Constantinescu et al., 2016). It is unclear whether this activity in different brain regions reflects the same underlying process, or different types of information. Future studies in humans using different experimental paradigms and different imaging resolutions will help to elucidate whether the grid code is homogenous across the brain, or shows functional specialization.

Reproducing the grid code analyses from previous studies is a time-consuming and non-trivial endeavor that involves advanced computer programming and mathematical skills. The GridCAT, therefore, opens up this cutting-edge research area to researchers less comfortable with programming by allowing users to analyze data using a GUI. Because the open-source code for the GridCAT is available in the download, it can be adapted and extended as desired by the user. To do this, the user would need to be competent in Matlab programming skills (either in Matlab’s proprietary programming language, or in other Matlab-compatible programming languages such as C or Fortran), have a Matlab license, and download the freely available SPM12 toolbox. Extensively commented example scripts are delivered with the GridCAT’s open-source code that show how functions and algorithms are programmatically called in the course of the grid code analysis pipeline. Furthermore, they also demonstrate how new functions and algorithms can be added to the GridCAT.

Relative to other standard fMRI analysis software packages, the unique contribution of the GridCAT is that it provides the algorithms necessary to detect the grid code in the BOLD signal, and that it synthesizes analysis pipelines that have been used previously. Specifically, these include different ways in which the data can be partitioned for GLM1 and GLM2, using either the GridCAT’s automated, or a user-defined partitioning scheme. The GridCAT then automatically estimates voxel-wise orientations of the grid code from the BOLD signal. Using these orientation values, the magnitude of grid code response can be calculated, as well as grid code metrics such as within- and between-voxel orientation coherence. Results can be visualized by using specific plotting tools offered by the GridCAT. Furthermore, it offers the option to statistically test for non-uniformity or clustering of voxel-wise grid orientation data, which has been used in previous grid code publications but is not commonly included in standard statistical software packages. Moreover, all data generated by the GridCAT can be exported using its data export tools, providing flexibility in terms of further statistical testing, comparisons and visualization, depending on the individual research questions and the researcher’s needs.

As noted above, although it is beyond the scope of this article to compare the results of all different model selection parameters, we believe that this is an important goal for the field so that researchers will have a better idea as to the factors that aid detection of these signals in fMRI data. By making all of these options available to the user, and the wider neuroscience community, the GridCAT has provided the first step in achieving this goal and has the potential to accelerate grid code research in humans.

## License Statement

The GridCAT is openly available at the Neuroimaging Informatics Tools and Resources Clearinghouse (NITRC) and can be downloaded from: http://www.nitrc.org/projects/gridcat.

The GridCAT is free software and can be redistributed and/or modified under the terms of the GNU General Public License as published by the Free Software Foundation, either version 3 of the License, or (at your option) any later version. A copy of the GNU General Public License is distributed along with the GridCAT, or can be found online at: http://www.gnu.org/licenses/.

## Author Contributions

MS, JS and TW substantially contributed to the conception and design of the work; conceptualized the toolbox; interpreted the data; gave final approval of the manuscript and agree to be accountable for all aspects of the work. MS implemented and designed the toolbox. MS and JS acquired and analyzed the data; drafted the work and TW revised it critically for important intellectual content.

## Conflict of Interest Statement

The authors declare that the research was conducted in the absence of any commercial or financial relationships that could be construed as a potential conflict of interest.

## Footnotes

^1^http://www.fil.ion.ucl.ac.uk/spm/

^2^http://www.itksnap.org/
